# Quantitative Modeling of the High-Throughput Production and *In Vivo* Kinetics of (Drug-Encapsulating) Liposomes

**DOI:** 10.1371/journal.pone.0010280

**Published:** 2010-04-23

**Authors:** Albert Wong

**Affiliations:** Program in Biological and Biomedical Sciences, Yale University, New Haven, Connecticut, United States of America; Tufts University, United States of America

## Abstract

In developing liposomes for *in vivo* use, it is important to design the liposomes to have optimal *in vivo* kinetics, and it is also necessary to identify optimal high-throughput production conditions for these liposomes. Previous work has not definitively established the general relationship between liposomes' configuration and composition, and their *in vivo* kinetics. Also, no straightforward method exists to calculate optimal liposome high-throughput production conditions for specific liposome compositions. This work presents first-principles quantitative correlations describing liposomes' *in vivo* drug leakage and vascular mass transfer kinetics. This work further presents a simple quantitative model relating specific liposome compositions to ideal high-throughput production parameters. The results have implications for the identification of promising liposome compositions via high-throughput screening methodologies, as well as the design and optimization of high-throughput reactors for liposome production.

## Introduction

Many drugs are attendant with significant systemic risks and side effects. To allow these drugs to achieve the treatment ideality of maximal efficacy and maximal specificity, it is necessary to use a targeted drug carrier to deliver and release the drugs specifically at the right time and at the right location [1].

Closed phospholipid vesicles (i.e., liposomes) are widely used as targeted drug carriers to deliver and release drugs in appropriate amounts at specific times and specific locations in the body. Existing liposomes exhibit undesirable *in vivo* characteristics including intrinsic destabilization, drug leakage, immunogenicity, and short plasma half-life [2]. Hence, research toward developing better liposomes is of significant importance.

Liposomes with optimal *in vivo* characteristics can be developed with knowledge of potential liposome compositions' *in vivo* kinetics. The *in vivo* kinetics of some liposome compositions have been characterized by experimental studies [3]. However, for rapid high-throughput screening of potential liposome compositions, it would be ideal to have broadly valid correlations allowing the prediction of liposomes' *in vivo* kinetics for many different liposome compositions.

Also, it is important to identify optimal high-throughput industrial liposome production conditions for liposome compositions of interest. While efficient industrial production conditions have been experimentally identified (mostly through trial and error) for some liposome compositions [3], it would be ideal to have a broadly valid quantitative model allowing the prediction of optimal production conditions for many different liposome compositions.

This work presents generally valid quantitative models describing liposomes' high-throughput production and predicting liposomes' *in vivo* drug leakage and vascular mass transfer kinetics for many liposome compositions.

## Results

### Encapsulated drug leakage from multilamellar liposome vesicles (MLVs)

**Figure 1:**
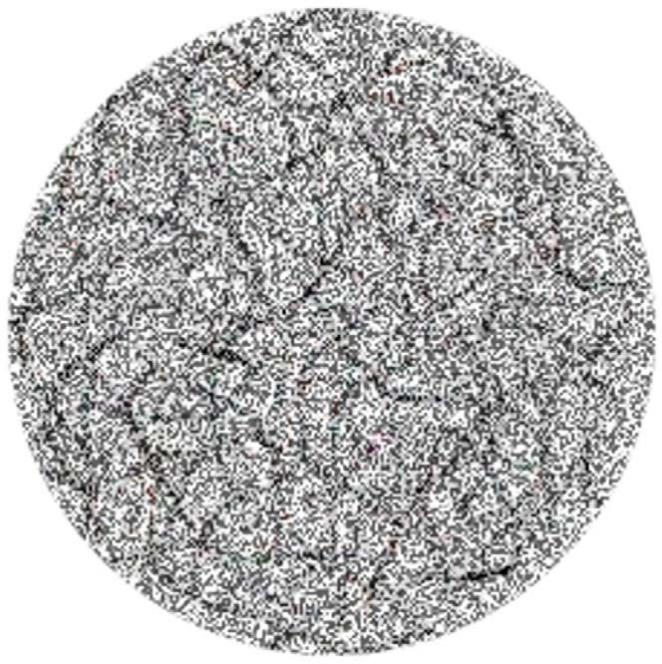
Encapsulated drug leakage from a MLV (schematic). A homogeneous, spherically symmetric, drug-encapsulating MLV.

Drug leakage from a widely used type of liposome, multilamellar liposomes (MLVs), is inherently minimized relative to drug leakage from one-layer, or unilamellar, liposomes due to MLVs' basic configuration (Figure 1 – artist's rendition of a cross section of a spherically symmetric, homogeneous, drug-encapsulating MLV). Via the general species conservation equation [4], drug leakage over time from a spherically symmetric, homogeneous, drug-encapsulating MLV can be described by the following series:
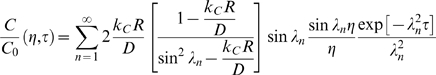
where:
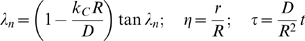



*C* = concentration of a given drug in a MLV

*C*_0_ = initial (immediate post-loading) concentration of the drug in the MLV

*D* = diffusivity of the drug in the MLV

*k_C_* = mass transfer coefficient of the drug

*R* = radius of the MLV

*t* = elapsed time

Here, the term “homogeneous” is used to refer to the drug particles being evenly mixed with the lipid molecules throughout the MLV.

### Vascular mass transfer

**Figure 2:**
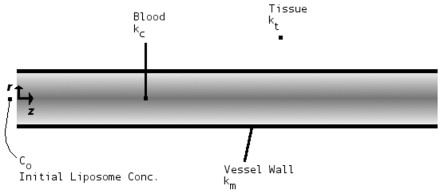
Vascular mass transfer of liposomes (schematic). Migration of liposomes in a blood vessel. Applicable mass transfer coefficients are shown.

A critically important issue associated with using liposomes in biological systems is the question of how the liposomes migrate in the bloodstream (Figure 2). From the general linear momentum conservation equation [4]–[6], the mass transfer of liposomes (of any type) in a blood vessel as a function of axial position can be described as:
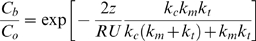
where:
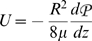



*C_b_* = bulk liposome concentration in a vessel

*C_o_* = initial (inlet) liposome concentration

*k_c_* = mass transfer coefficient of the liposomes in the bloodstream

*k_m_* = mass transfer coefficient of the liposomes in the vessel wall

*k_t_* = mass transfer coefficient of the liposomes in the tissue space


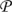
 = dynamic pressure in the vessel

*R* = radius of the blood vessel

*μ* = dynamic viscosity of the blood

*U* = mean velocity of fluid flow in the vessel

### High-throughput continuous tubular reactor (CTR) production rate

**Figure 3:**
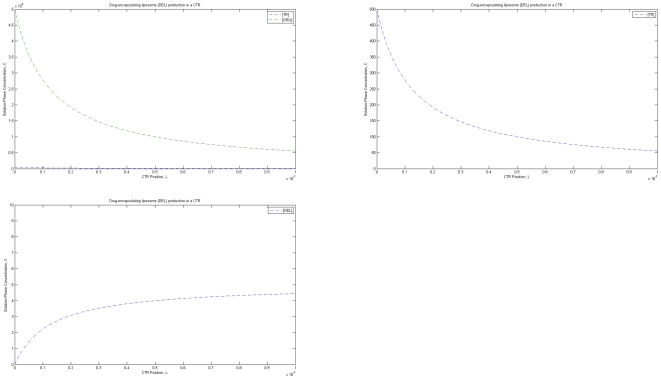
Production of liposomes in a CTR. Predicted concentrations of reagents and drug-encapsulating liposomes as a function of reactor position. Initial conditions used: [TR] = 500 arbitrary units (a.u.); [DRG] = 50000 a.u.; [DEL] = 0 a.u.

A highly efficient method for producing large quantities of drug-encapsulating liposomes rapidly comprises using a continuous flow reactor that can be run indefinitely as long as adequate quantities of reagents are supplied [7]. For these CTRs, a mole balance equation [8] can be used to develop a set of differential equations governing the liposome synthesis reaction. For example, for drug-encapsulating liposomes with targeting moieties (e.g., liposome-surface receptors), the synthesis reaction can be represented as follows, based on experimental measurements [9]–[11] of typical numbers of targeting moieties/receptors, drug particles, and lipid molecules per liposome (Figure 3):
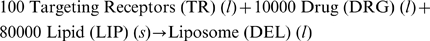
Assuming pseudo-second order kinetics with a large excess of lipid (LIP) [9]–[11], the governing differential equations are:
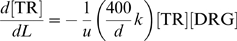


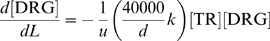


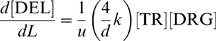
where:

*d* = CTR diameter

*k* = reaction rate coefficient

*u* = feed flow rate in the CTR

## Discussion

This work presents first principles quantitative correlations characterizing the core rate processes associated with the high-throughput production and *in vivo* kinetics of (drug-encapsulating) liposomes. Specifically, the models describe liposomes' encapsulated drug leakage kinetics, vascular mass transfer kinetics and high-throughput production kinetics.

The models can be used to facilitate the high-throughput screening of drug-encapsulating liposome compositions, configurations, and/or synthesis methods, with modeling data output employed as a preliminary rapid and low-cost filter in evaluating many different drug-encapsulating liposome compositions, configurations, and synthesis procedures. For example, the models could be used to screen different drugs to see which drugs, based on known biophysical properties, could potentially be carried and delivered effectively by a MLV. Also, the models could be used to screen different lipid modifications to see what kinds of modifications (based on known biophysical properties) might minimize undesired leakage of a particular drug. Other applications are also possible.

Compositions, configurations, or synthesis methods deemed to be promising based on the modeling output could then be further tested and characterized experimentally. Such a screening methodology is particularly important because past efforts to identify effective novel compositions, configurations, or synthesis methods have been limited due to an almost exclusive reliance on direct experimental testing. High-throughput screening using these models hence provides a way to test many more compositions, configurations, and synthesis methods (and identify more promising candidates) than experimental testing alone.

Further work may be performed to test and adapt these models for specific experimental conditions in practice. Also, future studies could extend these models to cover additional classes of liposomes and to other biologically relevant micro- and nanoparticles.

## Methods

### Species conservation



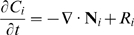
Where:

*C_i_* = molar concentration of species *i*

**N** *_i_* = molar flux of species *i* (using fixed coordinates)

*R_i_* = net rate of formation of species *i* per unit volume

Assume *i* is a minor component in a pseudobinary, constant density, constant diffusivity liquid solution. Then:
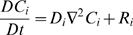
With no net rate of formation of species *i*, and no flow:
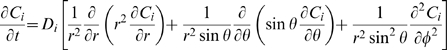
For a spherically symmetric, homogeneous drug-encapsulating MLV:
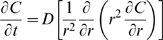
Where:

*C* = concentration of the drug within the MLV

*D* = diffusivity of the drug within the MLV

*t* = elapsed time

Boundary conditions are:
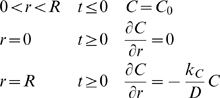
Where:

*k_C_* = mass transfer coefficient of the drug within the MLV

Scale and nondimensionalize:
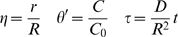


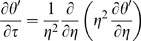


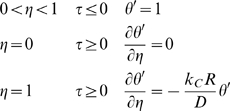
For ease of solution, transform:
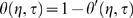


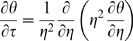


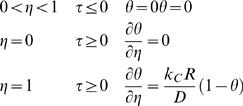
Apply FFT to solve, seeking solution of the form:
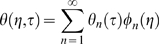
With basis functions:
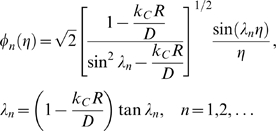
Transform:
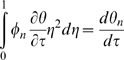


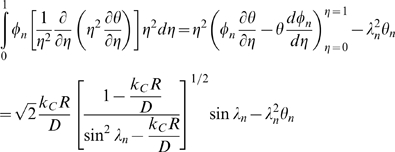


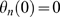
Hence:
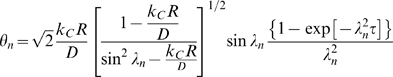
And:
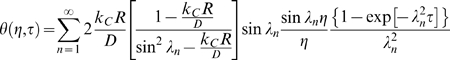
Which can be simplified upon inspection to:
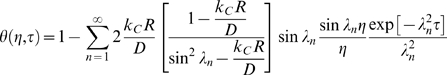
Therefore, the final solution is:
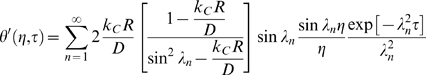


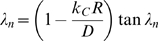


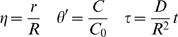



### Linear momentum conservation



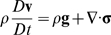
For a constant viscosity, constant density, incompressible Newtonian fluid:
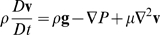
Or:
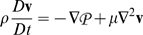
Where:


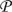
 = dynamic pressure

Assume fully developed unidirectional flow. Then:
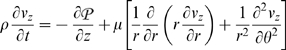
Further assume steady axisymmetric flow. Then:
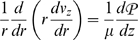
Integrate and apply symmetry condition at *r* = 0:
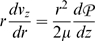
Integrate again and apply no-slip condition at *r* = *R* (*R* = vessel radius):
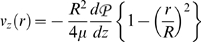
Or:
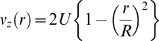


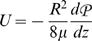
Per species conservation:
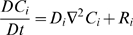
Assume large Péclet number (axial diffusion negligible). Then:
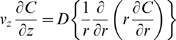
Hence:
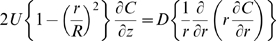
Where:

*C* = liposome concentration within the vessel

Integrate over *r*:
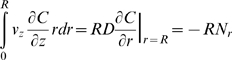
Where:

*N_r_* = liposome flux

Also:
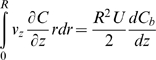
Where:
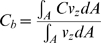



*A* = cross-sectional area of the vessel

Apply the following correlations:
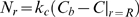


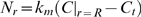


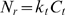
Where:

*C_t_* = liposome concentration in the tissue at the surface of the outer vessel wall

Then:
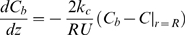
And:
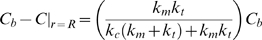
Therefore:
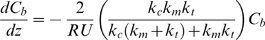


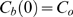
Hence, the final solution is:
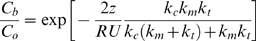


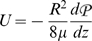



### CTR mole balance



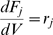
Assume low feed flow rate. Then:
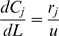
Where:

*C_j_* = concentration of species *j*

*L* = position in reactor

*u* = feed flow rate

Assume constant reactor radius and constant flow. Then:
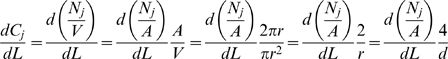
Where:

*A* = area of reactor

*d* = diameter of reactor

*N_j_* = number of moles of species *j*

*r* = radius of reactor

*V* = volume of reactor

Assume pseudo-second order kinetics with large excess of deposited lipid. Use:

100 Targeting Receptors (TR) (*l*)+10000 Drug (DRG) (*l*)+80000 Lipid (LIP) (*s*)→Liposome (DEL) (*l*)

Then:
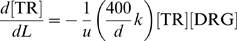


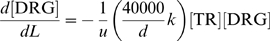


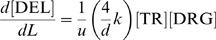
Where:

*d* = CTR diameter

*k* = reaction rate coefficient

*u* = feed flow rate in the CTR
